# Predicting the risk of colorectal cancer among diabetes patients using a random survival forest-guided approach

**DOI:** 10.3389/fonc.2024.1457446

**Published:** 2024-09-30

**Authors:** Sarah Tsz Yui Yau, Chi Tim Hung, Eman Yee Man Leung, Ka Chun Chong, Albert Lee, Eng Kiong Yeoh

**Affiliations:** JC School of Public Health and Primary Care, The Chinese University of Hong Kong, Hong Kong, Hong Kong SAR, China

**Keywords:** colorectal cancer, diabetes, risk prediction, survival analysis, random forest

## Abstract

**Background:**

Colorectal cancer (CRC) is the third most frequently diagnosed cancer worldwide. Diabetes and CRC share many overlapping lifestyle risk factors such as obesity, heavy alcohol use, and diet. This study aims to develop a risk scoring system for CRC prediction among diabetes patients using routine medical records.

**Methods:**

A retrospective cohort study was conducted using electronic health records of Hong Kong. Patients who received diabetes care in public general outpatient clinics between 2010 and 2019 and had no cancer history were identified, and followed up until December 2019. The outcome was diagnosis of CRC during follow-up. For model building, predictors were first selected using random survival forest, and weights were subsequently assigned to selected predictors using Cox regression.

**Results:**

Of the 386,325 patients identified, 4,199 patients developed CRC during a median follow-up of 6.2 years. The overall incidence rate of CRC was 1.93 per 1000 person-years. In the final scoring system, age, waist-to-hip ratio, and serum creatinine were included as predictors. The C-index on test set was 0.651 (95%CI: 0.631-0.669). Elevated serum creatinine (≥127 µmol/L) could be a potential important predictor of increased CRC risk.

**Conclusion:**

While obesity is a well-known risk factor for CRC, renal dysfunction could be potentially linked to an elevated risk of CRC among diabetes patients. Further studies are warranted to explore whether renal function could be a potential parameter to guide screening recommendation for diabetes patients.

## Introduction

Globally, colorectal cancer (CRC) ranks third in cancer incidence and second in cancer mortality (1). At the ecological level, CRC incidence is positively correlated with socioeconomic development, as indicated by human development index (2, 3).

Prior research has shown that CRC is associated with a number of lifestyle factors (3). Obesity (4), heavy alcohol use (5), Western dietary pattern (6), and processed meat (7) are established risk factors for CRC. On the other hand, physical activity (8) and long-term aspirin use (9) have been found to be protective against CRC. Some evidence also suggests that whole grain and calcium supplement are associated with a lower risk of CRC (3).

Numerous prediction or risk scoring models for CRC in the general population exist (10). Variables in these models included demographics (age and sex), behavioral factors (smoking and alcohol use), body mass index (BMI), medical history (cardiovascular disease, diabetes, and hypertension), medication use (aspirin and non-steroidal anti-inflammatory drugs), biomarkers (fasting glucose, cholesterol, and triglycerides), and dietary factors (10).

Nevertheless, CRC is linked to many lifestyle factors which may not be available in routinely collected data (10). Moreover, increasing the number of variables or model complexity may not necessarily improve performance (10). Furthermore, reliance on traditional univariate regression in variable selection may omit potential influential predictors (11). In addition, diabetes and CRC share many overlapping risk factors. Previous epidemiological studies have shown that patients with obesity (12), heavy alcohol use (13), or poor diet (14) are more likely to develop diabetes. Heavy alcohol use or poor diet is often linked to excess caloric intake, which in turn potentially promotes obesity. One potential underlying pathophysiological mechanism linking diabetes and CRC is adipose tissue dysfunction in obesity leading to insulin resistance, diabetes, and metabolic dysfunction (15), characterized by a chronic state of low-grade inflammation, which in turn promotes carcinogenesis (16). Given many common risk factors shared between diabetes and CRC, patients with diabetes are more likely to be diagnosed with CRC than the general population (17). However, there is a lack of risk prediction models for CRC among diabetes patients.

While traditional regression approach has been adopted in building CRC prediction models, machine learning approach such as tree-structured algorithms (18) and neural network (19) have also been applied (20). Nevertheless, the lack of interpretability may hinder its application (20). Recently, an interpretable machine learning framework to develop clinical scoring system has been proposed (21), where variable selection is guided by random survival forest, and weight assignment is performed using conventional Cox regression. The advantages of the framework include: i) tree-structured algorithms are more suitable for handling non-linear relationships between covariates and an outcome as well as capturing interactions among covariates on an outcome; ii) an ensemble tree algorithm reduces variance in prediction; iii) a less biased approach has been adopted in selecting less established predictors; iv) Cox regression remains the most widely accepted approach in developing risk scoring models for time-to-event outcomes; and v) clinical expert knowledge is incorporated in risk score development.

To fill the gaps in the literature on i) the lack of CRC prediction models among asymptomatic general population based on solely routine medical records; ii) the lack of individualized prediction models among diabetes population; and iii) the lack of interpretability in machine learning approach, this study aims to i) develop a parsimonious scoring system for CRC prediction among diabetes patients based on electronic health records; and ii) identify potential parameters to guide CRC screening recommendation for diabetes patients using a random survival forest-guided approach.

## Methods

### Study design and study population

This is a retrospective cohort study based on territory-wide electronic health records of Hong Kong’s public healthcare system. The Hospital Authority (HA) is a statutory body managing 43 public hospitals, 49 specialist outpatient clinics, and 74 general outpatient clinics. The Hong Kong population are largely homogenous ethnic Chinese (over 95%). The HA maintains a centralized clinical data repository to store information on patients’ demographics, prescription records, disease diagnoses, inpatient admissions, outpatient attendances, and laboratory results. Disease diagnoses were coded according to the International Classification of Disease 9^th^ or 10^th^ revision (ICD-9 or ICD-10), or the International Classification of Primary Care 2^nd^ edition (ICPC-2). Data were accessed via HA Data Collaboration Lab. Ethics approval for secondary data analysis was provided by the Joint Chinese University of Hong Kong-New Territories East Cluster Clinical Research Ethics Committee.

### Patients

Patients who received diabetes care at any of the general outpatient clinics between 2010 and 2019 were initially included. Those who i) were diagnosed with non-type 2 diabetes; ii) were diagnosed with diabetes below the age of 18 years; iii) had a history of malignancy prior to a baseline Diabetes Mellitus Complication Screening (DMCS) assessment; or iv) had a follow-up period of less than six months were excluded. Patients were followed up until a CRC diagnosis, death, or December 2019, whichever was earlier.

### Outcome

The outcome of interest was diagnosis of CRC (ICD-9: 153-154; ICD-10: C18-21) during follow-up.

### Input variables

Input variables were information ascertained during a baseline DMCS assessment. Variables included demographics (sex and age), duration of diabetes, medical history (ischemic heart disease, cerebrovascular disease, heart failure, hypertension, chronic kidney disease, liver cirrhosis, chronic obstructive pulmonary disease, pneumonia, and family history of diabetes), medication use (anti-diabetic drugs: metformin, sulfonylurea, insulin, and dipeptidyl peptidase-4 inhibitors, aspirin, nonsteroidal anti-inflammatory drugs, anti-coagulants, anti-platelets, anti-hypertensive drugs, and statins), behavioral factors (alcohol use and smoking), anthropometric measurements (BMI and waist-to-hip ratio), and laboratory measurements (serum creatinine, HbA_1c_, fasting glucose, low-density lipoprotein cholesterol, high-density lipoprotein cholesterol, and triglycerides). Medication use was coded as dichotomous variables indicating whether patients had been prescribed a drug at the time of the assessment. Laboratory measurements were taken from latest results to the date of assessment.

### Data analysis

To balance class distribution and maintain a sufficient sample size, patients who developed CRC (n=4,199) and a random subset of patients who did not develop CRC during follow-up (n=41,990) were selected for model building in a 1:10 ratio. Patients were randomly split into training, validation, and test set in a 7:1:2 ratio by default. The set of input variables was ranked by their relative variable importance to CRC prediction on training set by random survival forest algorithm. Each variable was then sequentially added to the scoring model according to the ranking until no further model improvement on validation set was shown. A final set of predictors of the scoring model was then selected using model improvement and model parsimony as criteria. A weight was assigned to each predictor with reference to the lowest beta coefficient of all variables in the scoring model using Cox regression. The number of trees in the random survival forest model was set as 30. For continuous variables, the cutoffs were set by default quantiles. The CRC-free survival probability of patients by score was assessed using Kaplan–Meier method. Model performance was evaluated using Harrell’s concordance (C-) index or area under the curve (AUC) as metrics. Data analyses were conducted using R software (version 4.2.3; R Foundation for Statistical Computing, Vienna, Austria).

## Results

Of the 386,325 diabetes patients identified, 4,199 patients developed CRC during a median follow-up of 6.2 years. Overall, the incidence rate of CRC among patients of both sexes was 1.93 per 1000 person-years, whereas the incidence rates among females and males were 1.58 and 2.30 per 1000 person-years respectively. Patients who were assigned a score of 80 to 100 tended to be older (mean: 72.9 vs 57.4 years, *p*<0.001), male (56.22 vs 48.49%, *p*<0.001), have an elevated waist-to-hip ratio (0.95 vs 0.93, *p*<0.001) and a higher serum creatinine (101.79 vs 72.17 µmol/L, *p*<0.001), when compared to those assigned a score below 80 (**Supplementary Table S1**).

### Final scoring system

Age, waist-to-ratio, and serum creatinine were identified as variables of highest importance to predict the risk of CRC. Sex was not identified among the top ten important variables.

In the final 100-point time-to-event scoring system, age, waist-to-hip ratio, and serum creatinine were assigned up to 76, 10, and 14 points respectively. In general, the risk of CRC started to increase from the age of 44 years onwards. The risk would almost double when patients reaching 53 years, and continue to rise until 82 years. On the other hand, waist-to-hip ratio appeared to be approximately positively associated with the risk of CRC. The risk of CRC rose noticeably from the ratio of 0.89 onwards, and continued to increase up to the ratio of 1.04 (**Table 1**). The increasing trend remained similar when controlling for sex (**Supplementary Table S2**). In addition, serum creatinine demonstrated a potential non-linear relationship with the risk of CRC. Serum creatinine appeared to be an important predictor of CRC risk, in particular when the level reaching 127 µmol/L or above (**Table 1**). The steep increase in weight from the level of 127 µmol/L or above was more obvious when controlled for sex (**Supplementary Table S2**). In the model with the addition of sex, waist-to-hip ratio ≥1.04, serum creatinine ≥127 µmol/L, and male sex carried similar weights in predicting CRC risk (**Supplementary Table S2**).

**Table 1:** Final scoring system for colorectal cancer prediction among diabetes patients.

Variable	Value	Point
Age, years	<44	0
	[44, 53)	33
	[53, 73)	59
	[73, 82)	73
	≥82	76
Waist-to-hip ratio	<0.84	0
	[0.84, 0.89)	2
	[0.89, 0.99)	6
	[0.99, 1.04)	8
	≥1.04	10
Serum creatinine, µmol/L	<51	6
	[51, 62)	0
	[62, 94)	10
	[94, 127)	12
	≥127	14

### CRC-free survival during follow-up

**Figure 1:**
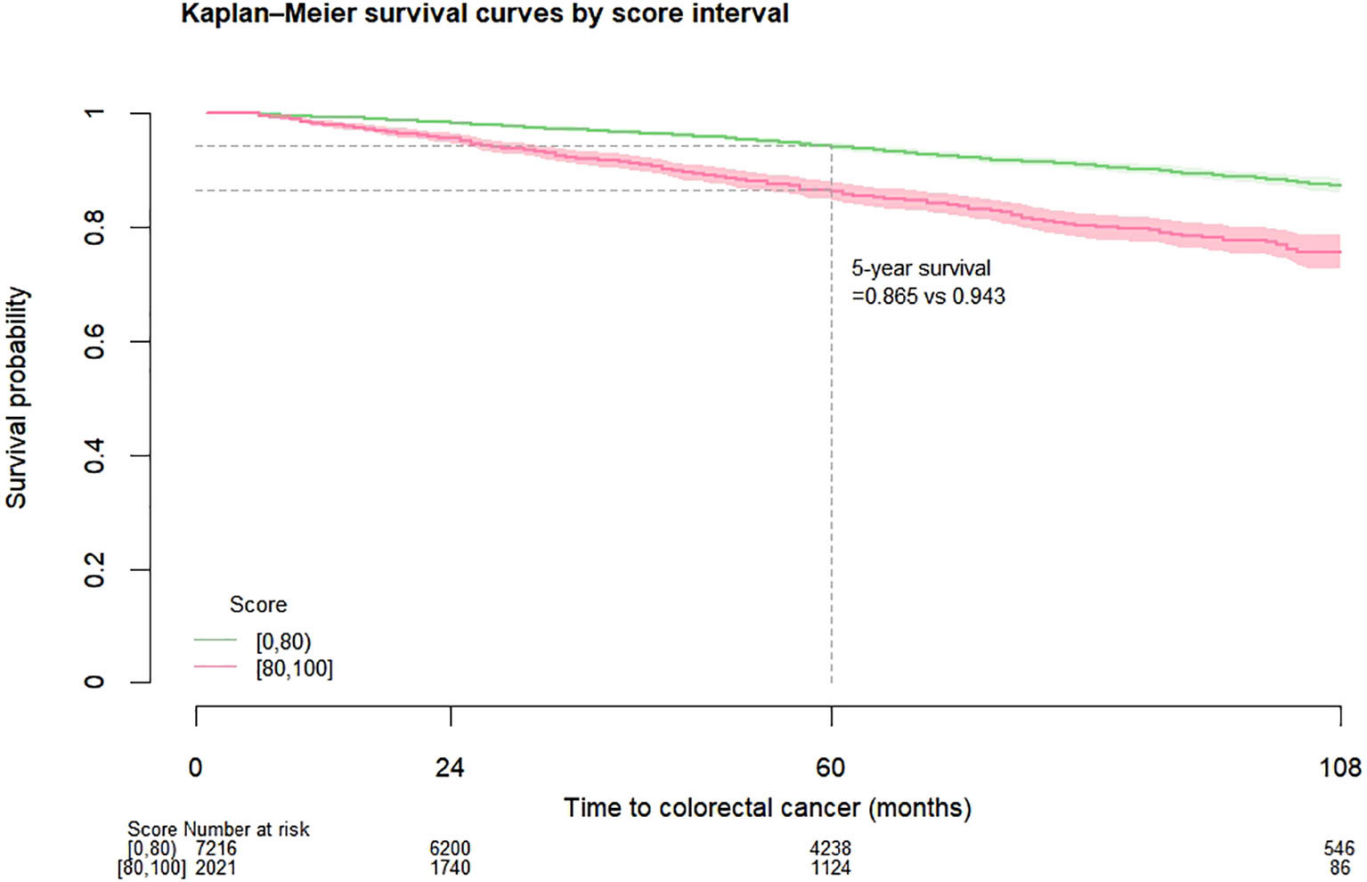
Kaplan-Meier colorectal cancer-free survival curves among diabetes patients on test set by risk score.

Among the entire cohort, for patients with score 0 to 79 and 80 to 100, the CRC-free survival probability at 5 years ranged from 0.994 to 0.985, and the corresponding probability at 7 years dropped to 0.990 and 0.978 (**Table 2**). The proportion of patients with highest score (90 to 100) who developed CRC was 2.08% (**Table 3**). **Figure 1** shows the CRC-free survival among patients on test set.

**Table 2:** Colorectal cancer-free survival probability of diabetes patients in the entire cohort at different follow-up time points by score interval.

Time	Score interval	
	[0, 79)	[80, 100]
t= 2 years	0.998	0.995
t= 5 years	0.994	0.985
t= 7 years	0.990	0.978
t= 9 years	0.986	0.972

**Table 3:** Distribution of proportion of diabetes patients in the entire cohort who developed colorectal cancer during follow-up by score interval.

Score interval	Number of patients, n	Number of patients who developed colorectal cancer during follow-up, n (%)	
[0, 10)	1,248	0	(0%)
[10, 20)	6,694	4	(0.06%)
[20, 30)	11,339	9	(0.08%)
[30, 40)	2,424	4	(0.17%)
[40, 50)	16,180	43	(0.27%)
[50, 60)	38,713	147	(0.38%)
[60, 70)	41,820	291	(0.70%)
[70, 80)	142,565	1,523	(1.07%)
[80, 90)	59,274	801	(1.35%)
[90, 100]	66,068	1,377	(2.08%)

### Model performance

The C-index of the final model on validation set was 0.644. When the model was applied to test set, the C-index was 0.651 (95% confidence interval [CI]: 0.631-0.669) At 2, 5, and 7 years, the AUCs were 0.622 (95%CI: 0.545-0.708), 0.588 (95%CI: 0.51-0.66), and 0.711 (95%CI: 0.577-0.834) respectively. When the model was applied to the full cohort, the C-index was 0.663 (95%CI: 0.655-0.671).

## Discussion

The current study applied random survival forest in variable selection to inform the subsequent development of CRC risk scoring among diabetes patients based on an earlier proposed framework (21). While variable ranking in random survival forest incorporates the aggregate results of the multidimensional relationships among covariates associated with CRC in individual survival trees, the subsequent scoring reduces the number of dimensions in representing both the main and interaction effects of important variables and condenses information into a simple score to potentially guide decision-making. Findings of the study demonstrated that obesity remained a strong predictor of CRC among the more homogeneous diabetes population. On the other hand, renal dysfunction, a potential complication of diabetes, could be a potential parameter to guide CRC screening among diabetes patients, who are at greater risk of developing CRC than the general population (17). Nevertheless, male sex and smoking were ranked as less important predictors in this study.

Among the risk scoring models for CRC prediction among asymptomatic general population, BMI has been incorporated as a predictor in several existing models. For example, Betes et al. (22) developed a simple score with only three predictors, namely age, sex, and BMI, to predict the risk of advanced adenomas among individuals aged 40 years or above in the absence of family history of CRC who underwent a colonoscopy. On the other hand, several studies (23–26) demonstrated that incorporating BMI as an additional predictor to the original Asia-Pacific Colorectal Screening (APCS) score (27), which included age, sex, family history of CRC, and smoking as predictors of colorectal advanced neoplasia among adults who received a colonoscopy, may potentially improve model performance.

The current study showed that waist-to-hip ratio could be a potential alternative predictor of CRC over BMI among diabetes patients who receive routine care in primary care clinics. While obesity is associated with an elevated risk of CRC, obesity is mainly measured by overall obesity (BMI) but less commonly by abdominal obesity indicators, such as waist-to-hip ratio and waist circumference (4). However, emerging evidence suggests that abdominal obesity could be more predictive of cancer risk than overall obesity (28). While BMI is a more practically convenient measure, waist circumference can be incorporated as an alternative measure when available.

The present study also found that serum creatinine 127 µmol/L or above could be a potential important indicator of elevated CRC risk among diabetes patients. In a multi-center retrospective cohort study performed in China, serum creatinine demonstrated a non-linear association with the risk of all-cause mortality among CRC patients (29). Patients with high serum creatinine level (>104 µmol/L for male or >85 µmol/L for female) had a shorter survival than those with serum creatinine falling within normal range (29). In another study performed in the Western population, elevated serum creatinine was only shown to be linked to a higher risk of all-cause mortality among patients with rectal cancer but not colon cancer (30). Nevertheless, despite serum creatinine being a potential prognostic marker of CRC (29, 30), existing literature on whether serum creatinine is a predictor of CRC development remains limited. Moreover, although serum creatinine is linked to both total muscle mass and dietary meat intake (31), dietary information was not available in the above two studies (29, 30). However, the latter study (30) investigated the associations between a large number of metabolites and mortality among CRC patients, where metabolites could be a reflection of dietary patterns (32), and found that only serum creatinine was associated with all-cause mortality after accounting for multiple comparisons. Furthermore, the changes in serum creatinine among patients who subsequently developed CRC could be due to disrupted intestinal microbial flora and altered creatinine metabolism over the course of CRC carcinogenesis (29, 33, 34).

On the other hand, elevated serum creatinine could be an indicator of renal dysfunction or severity of diabetes condition. While the relationship between renal dysfunction and CRC remains less conclusive, emerging studies suggest that renal dysfunction could be linked to an elevated risk of CRC (35–38). Possible mechanisms linking renal dysfunction to CRC could be chronic inflammation and oxidative stress (39). Nevertheless, different markers for renal function exist. Lees et al. (38) previously reported that cystatin C could be a more sensitive renal function indicator for cancer risk prediction. Future studies are warranted to examine the association between renal function and CRC risk, and whether serum creatinine is the most feasible and sensitive renal function indicator among diabetes population.

Compared to a simple score using routine medical data (10, 22) and (modified) APCS score for Asian population (24, 27), similar as the majority of CRC prediction models (10), these three models (22, 24, 27) targeted at asymptomatic general population who underwent a colonoscopy, while the current study targeted at diabetes patients who received routine diabetes care in primary care. Also, while Betes et al.’s score (22) was developed using routine medical data, (modified) APCS score was based on information from questionnaires (24, 27). These three models (22, 24, 27) yielded an AUC ranging from 0.65 to 0.67. The proposed score using routine medical data demonstrated a comparable moderate performance and could be potentially useful to inform risk stratification strategies and CRC screening guidelines for diabetes patients.

Findings of the present study imply that consistent with the current guidelines (40), diabetes patients in the study cohort demonstrated a mildly elevated risk of CRC starting from the age of 44 years onwards, and the risk rose markedly from 53 years old. Obesity remains a key predictor of CRC among the homogeneous diabetes population, regardless of the indicators used. While existing literature on the links between serum creatinine and the risk of CRC remains scarce, it is possible that serum creatinine or renal dysfunction could be a predictor of CRC among diabetes population. The potential clinical and public health implications of the study are i) to explore whether obesity or renal function indicator should be incorporated as additional parameters to guide screening recommendation for diabetes patients given that diabetes is linked to obesity and renal dysfunction; and ii) to examine whether improved renal function could potentially lower the risk of CRC among diabetes population.

There are several limitations of the present study. First, information on family history of CRC or dietary factors was not available in this study. Nevertheless, the proposed model only utilized routine medical records and does not require additional data collection (10). Second, serum creatinine is associated with dietary meat intake (31), however, dietary information is not available in routine medical records. The apparent observed association between serum creatinine and CRC risk could be confounded by the links between diet and CRC (6, 7). Nevertheless, only serum creatinine, but not other 147 metabolites, was found to be linked to an increased risk of all-cause mortality among CRC patients from four European cohorts (30). Third, renal function could be linked to liver function, however, liver function was not evaluated in this study. Fourth, chronic kidney disease was a dichotomous input variable in the present study. Further research on the severity of kidney disease on CRC risk would be warranted. Fifth, duration and dosage of medication use was not captured in the present study. Sixth, external validation was not available in this study, however, internal validation was conducted on the unseen test set. Finally, generalizability of the findings could be limited to Asian diabetes population.

## Conclusions

While abdominal obesity is a well-established risk factor for CRC, renal dysfunction could also be a potential parameter for CRC screening among diabetes patients based on routine medical records. Further studies are warranted to examine whether obesity or renal function could be potential additional criteria to guide CRC screening recommendation for diabetes patients.

## Data Availability

The datasets presented in this article are not readily available because data access is restricted. Requests to access the datasets should be directed to EL, yeemanleung@cuhk.edu.hk.
