# Supplementary material for: Predicting the risk of colorectal cancer among diabetes patients using a random survival forest-guided approach
Source: Front Oncol. 2024 Sep 30;14:1457446. doi: 10.3389/fonc.2024.1457446 (PMC11471444; doi:10.3389/fonc.2024.1457446)
Supplement: Supplementary file 1 [file Table1.docx]

Table S1. Characteristics of diabetes patients in the entire cohort by assigned score interval for colorectal cancer risk prediction.

|  | **Score <80** | | **Score 80 to 100** | |
| --- | --- | --- | --- | --- |
| **Characteristics** | **(n=260,983)** | | **(n=125,342)** | |
| Colorectal cancer cases during follow-up, n (%) | 2,021 | (0.77%) | 2,178 | (1.74%) |
| Demographics |  |  |  |  |
| Male, n (%) | 126,563 | (48.49%) | 70,463 | (56.22%) |
| Age at assessment in year, mean±SD | 57.4 | ±9.08 | 72.9 | ±9.04 |
| Duration of diabetes in year, median (IQR) | 2 | (0-7) | 6 | (1-12) |
| Medical history |  |  |  |  |
| Ischemic heart disease, n (%) | 12,910 | (4.95%) | 15,229 | (12.15%) |
| Cerebrovascular disease, n (%) | 10,669 | (4.09%) | 13,290 | (10.60%) |
| Heart failure, n (%) | 2,492 | (0.95%) | 5,355 | (4.27%) |
| Hypertension, n (%) | 212,054 | (81.25%) | 116,630 | (93.05%) |
| Chronic kidney disease, n (%) | 33,563 | (12.86%) | 25,151 | (20.07%) |
| Liver cirrhosis, n (%) | 5,508 | (2.11%) | 2,157 | (1.72%) |
| Chronic obstructive pulmonary disease, n (%) | 901 | (0.35%) | 1,915 | (1.53%) |
| Pneumonia, n (%) | 5,186 | (1.99%) | 6,923 | (5.52%) |
| Family history of diabetes, n (%) | 138,984 | (53.25%) | 43,229 | (34.49%) |
| Medication use |  |  |  |  |
| Anti-diabetic drugs |  |  |  |  |
| Metformin, n (%) | 108,319 | (41.50%) | 48,834 | (38.96%) |
| Sulfonylurea, n (%) | 66,851 | (25.62%) | 38,234 | (30.50%) |
| Insulin, n (%) | 14,234 | (5.45%) | 9,815 | (7.83%) |
| Dipeptidyl peptidase-4 inhibitors, n (%) | 8,730 | (3.35%) | 6,095 | (4.86%) |
| Sodium-glucose cotransporter-2 inhibitors, n (%) | 796 | (0.31%) | 247 | (0.20%) |
| Glucagon-like peptide-1 receptor agonists, n (%) | 163 | (0.06%) | 34 | (0.03%) |
| Glucosidase inhibitor, n (%) | 952 | (0.36%) | 622 | (0.50%) |
| Glitazone, n (%) | 97 | (0.04%) | 50 | (0.04%) |
| Meglitinide, n (%) | 999 | (0.38%) | 428 | (0.34%) |
| Aspirin, n (%) | 39,611 | (15.18%) | 40,965 | (32.68%) |
| Nonsteroidal anti-inflammatory drugs, n (%) | 142,759 | (54.70%) | 65,274 | (52.08%) |
| Anti-coagulants, n (%) | 9,148 | (3.51%) | 9,886 | (7.89%) |
| Anti-platelets, n (%) | 14,567 | (5.58%) | 13,152 | (10.49%) |
| Anti-hypertensive drugs, n (%) | 165,097 | (63.26%) | 100,406 | (80.11%) |
| Statins, n (%) | 122,854 | (47.07%) | 69,165 | (55.18%) |
| Behavioral factors |  |  |  |  |
| Current drinker/ ex-drinker, n (%) | 61,749 | (23.66%) | 19,908 | (15.88%) |
| Current smoker/ ex-smoker, n (%) | 74,660 | (28.61%) | 41,318 | (32.96%) |
| Anthropometric measurements |  |  |  |  |
| Body mass index in kg/m^2^, mean±SD | 26.28 | ±4.39 | 25.64 | ±3.67 |
| Waist-to-hip ratio, mean±SD | 0.93 | ±0.06 | 0.95 | ±0.06 |
| Laboratory measurements |  |  |  |  |
| Serum creatinine in µmol/L, mean±SD | 72.17 | ±26.61 | 101.79 | ±56.93 |
| HbA_1c_ in %, mean±SD | 7.44 | ±1.53 | 7.20 | ±1.26 |
| Fasting glucose in mmol/L, mean±SD | 7.75 | ±2.35 | 7.35 | ±2.02 |
| Low-density lipoprotein cholesterol in mmol/L, mean±SD | 2.72 | ±0.84 | 2.58 | ±0.80 |
| High-density lipoprotein cholesterol in mmol/L, mean±SD | 1.27 | ±0.33 | 1.26 | ±0.33 |
| Triglycerides in mmol/L, mean±SD | 1.64 | ±1.26 | 1.56 | ±0.99 |

Table S2. Scoring system for colorectal cancer prediction among diabetes patients with the addition of sex.

| **Variable** | **Value** | **Point** |
| --- | --- | --- |
| Age, years | <44 | 0 |
|  | [44, 53) | 32 |
|  | [53, 73) | 60 |
|  | [73, 82) | 74 |
|  | ≥82 | 76 |
| Waist-to-hip ratio | <0.84 | 0 |
|  | [0.84, 0.89) | 1 |
|  | [0.89, 0.99) | 3 |
|  | [0.99, 1.04) | 5 |
|  | ≥1.04 | 8 |
| Serum creatinine, µmol/L | <51 | 5 |
|  | [51, 62) | 0 |
|  | [62, 127) | 5 |
|  | ≥127 | 8 |
| Sex | Female | 0 |
|  | Male | 9 |

Table S3. Distribution of proportion of diabetes patients on test set who developed colorectal cancer during follow-up by score interval.

| **Score interval** | **Number of patients, n** | **Number of patients who developed colorectal cancer during follow-up, n (%)** | |
| --- | --- | --- | --- |
| [0, 10) | 115 | 0 | (0%) |
| [10, 20) | 316 | 2 | (0.63%) |
| [20, 30) | 26 | 0 | (0%) |
| [30, 40) | 226 | 4 | (1.77%) |
| [40, 50) | 711 | 25 | (3.52%) |
| [50, 60) | 380 | 22 | (5.79%) |
| [60, 70) | 967 | 62 | (6.41%) |
| [70, 80) | 4,475 | 411 | (9.18%) |
| [80, 90) | 927 | 134 | (14.46%) |
| [90, 100] | 1,094 | 183 | (16.73%) |
